# Implementation of the Mental Capacity Act: a national observational study comparing resultant trends in place of death for older heart failure decedents with or without comorbid dementia

**DOI:** 10.1186/s12916-021-02210-2

**Published:** 2022-01-20

**Authors:** James M. Beattie, Irene J. Higginson, Theresa A. McDonagh, Wei Gao

**Affiliations:** 1 Cicely Saunders Institute of Palliative Care and Rehabilitation, King’s College London, London, UK; 2 School of Cardiovascular Medicine and Sciences, King’s College London, London, UK

**Keywords:** Heart failure, Dementia, Place of death, Mental Capacity Act, Comorbidity

## Abstract

**Background:**

Heart failure (HF) is increasingly prevalent in the growing elderly population and commonly associated with cognitive impairment. We compared trends in place of death (PoD) of HF patients with/without comorbid dementia around the implementation period of the Mental Capacity Act (MCA) in October 2007, this legislation supporting patient-centred decision making for those with reduced agency.

**Methods:**

Analyses of death certification data for England between January 2001 and December 2018, describing the PoD and sociodemographic characteristics of all people ≥ 65 years registered with HF as the underlying cause of death, with/without a mention of comorbid dementia. We used modified Poisson regression with robust error variance to determine the prevalence ratio (PR) of the outcome in dying at home, in care homes or hospices compared to dying in hospital. Covariates included year of death, age, gender, marital status, comorbidity burden, index of multiple deprivation and urban/rural settings.

**Results:**

One hundred twenty thousand sixty-eight HF-related death records were included of which 8199 mentioned dementia as a contributory cause. The overall prevalence proportion of dementia was 6.8%, the trend significantly increasing from 5.6 to 8.0% pre- and post-MCA (Cochran-Armitage trend test *p* < 0.0001). Dementia was coded as unspecified (78.2%), Alzheimer’s disease (13.5%) and vascular (8.3%). Demented decedents were commonly older, female, and with more comorbidities. Pre-MCA, PoD for non-demented HF patients was hospital 68.2%, care homes 20.2% and 10.7% dying at home. Corresponding figures for those with comorbid dementia were 47.6%, 48.0% and 4.2%, respectively. Following MCA enforcement, PoD for those without dementia shifted from hospital to home, 62.5% and 17.2%, respectively; PR: 1.026 [95%CI: 1.024–1.029]. While home deaths also rose to 10.0% for those with dementia, with hospital deaths increasing to 50.4%, this trend was insignificant, PR: 1.001 [0.988–1.015]. Care home deaths reduced for all, with/without dementia, PR: 0.959 [0.949–0.969] and PR: 0.996 [0.993–0.998], respectively. Hospice as PoD was rare for both groups with no appreciable change over the study period.

**Conclusions:**

Our analyses suggest the MCA did not materially affect the PoD of HF decedents with comorbid dementia, likely reflecting difficulties implementing this legislation in real-life clinical practice.

**Supplementary Information:**

The online version contains supplementary material available at 10.1186/s12916-021-02210-2.

## Background

The incidence and prevalence of heart failure varies across the world reflecting regional differences in cardiovascular disease burden, ethnic and socioeconomic diversity. While the age-adjusted incidence and prevalence of heart failure may be declining in Westernised countries, absolute rates of these indices are increasing in parallel with societal ageing [1]. In the United Kingdom (UK), about 900,000 people are living with heart failure. The prevalence is 1–2% in the general population, rising to at least 10% in those ≥70 years of age [2, 3]. The lifetime risk of developing heart failure is about 20% at 40 years, but for each age decile between 65 and 85 years, the incidence doubles for men and trebles for women [4].

Crafted by international consensus [5], a universal definition of heart failure has recently emerged as

…a clinical syndrome with symptoms and / or signs caused by a structural and / or functional cardiac abnormality and corroborated by elevated natriuretic peptide levels and / or objective evidence of pulmonary or systemic congestion.

Increasingly accurate diagnostic protocols have been established, and based on the left ventricular ejection fraction (EF), the percentage volume of the diastolic blood pool ejected during systole, an updated classification system linked to the above definition has characterised four clinical phenotypes: heart failure with a reduced EF [< 40%] (HFrEF); heart failure with a mildly reduced EF [40–49%] (HFmrEF); heart failure with a preserved EF [≥50%] (HFpEF); and heart failure with an improved EF [baseline EF ≤40%, a ≥10% point increase from baseline EF, the improved EF > 40%] (HFimpEF) [5]. Advances in heart failure therapy, particularly those for HFrEF, have enabled some patients to live longer, more comfortable lives, but for many, heart failure remains a life-limiting condition, the 5- and 10-year case-fatality rates being about 50% and 75%, respectively, similar to outcomes for common cancers [3, 6]. As well as being encumbered with an unpredictable disease trajectory, this burgeoning and increasingly elderly clinical cohort usually exhibit several comorbidities which add to the complexity and challenge the coordination required of their care [2].

Cognitive impairment, ranging from mild forms to severe as manifest in dementia, is relatively common, affecting between 25 and 70% of those with heart failure across a series of studies, and estimated at 40% overall in a meta-analysis [7–9]. Disordered cognition is heterogeneous and demonstrable across a range of higher cortical domains including attention, memory, speech and language processing, learning and executive function [10], such deficits beyond those arising from normative ageing of the brain. Cognitive impairment may be transitory, sometimes occurring as delirium in patients presenting with acute heart failure [11], but often presents as a long-term progressive condition, more frequently encountered in older people with chronic heart failure compared to their age-matched healthy counterparts [12]. The impact of these persistent features tends to fluctuate over time, but even when mild, may impact heart failure patients’ self-care behaviours and treatment adherence resulting in greater rates of hospital admission and mortality [13, 14]. There appears to be no direct correlation between the severity of these two conditions [15], but dilemmas may arise when treating cognitively impaired heart failure patients, sometimes relating to the continued efficacy of established treatment modalities, ceilings of care, and resuscitation issues [16]. These confront not only professional healthcare providers but also their informal carers, usually family members, who take responsibility for much day-to-day practical support. In assisting those affected by dementia-related loss of intellectual capacity, such carers may be called upon to act as decisional proxies and offer insight into patients previously voiced values and preferences for treatment.

The Mental Capacity Act (MCA) of 2005, applicable to residents in England and Wales aged ≥16 years, sets out a statutory framework to foster person-centred decision making and advance care planning for those who may lack capacity due to a lifelong learning disability, or as a consequence of the transient or permanent effects of acute or long-term illnesses [17]. For people with cognitive impairment and a progressive, ultimately fatal condition such as heart failure, the MCA may be particularly important in fulfilling goals of care close to the end of life. Achieving care in appropriate settings and the preferred place of death (PoD) are generally accepted as benchmarks of good quality end-of-life care, death at home or customary place of residence usually regarded as the desired option [18]. Given the relatively frequent association of cognitive impairment with heart failure, it might be expected that the decision-making processes legally constituted within the MCA would drive changes in the final place of care and death during the terminal phase of this condition. The code of practice setting out the standards required to comply with the MCA came fully into force on October 1, 2007 [19]. Thus, we undertook a comparative trend analysis covering the period of implementation of this legislation to discern any resultant variation in the PoD of heart failure decedents rendered vulnerable by comorbid dementia.

## Methods

### Application of the Mental Capacity Act

As outlined above, the MCA and associated code of practice offer legislative protection to promote patient empowerment and safeguard their autonomy. Prepared when mental capacity is intact, patients may formulate an advance decision such as one to refuse life-sustaining treatment or, if ≥18 years, appoint a close person as a personal welfare lasting power of attorney (LPA) to undertake decisions on their behalf if agency is later lost. Thereafter, any clinical treatment protocol, where possible, should be in accordance with their previously documented choices and values, or these as expressed through their nominated personal welfare LPA. For the purposes of the Act, a two-stage capacity test is applicable. To qualify through Stage 1, the individual must exhibit a demonstrable functional impairment of the mind or brain. For Stage 2, capacity is deemed to be lost if they lack the ability to fulfil any of the following: (a) understand the information pertinent to the decision, (b) retain the information, (c) deliberate on that information as part of the decision-making process and (d) communicate their decision by any means possible. In the context of the study, we must emphasise that many people diagnosed with dementia can still make decisions about many aspects of their care, and loss of capacity should not be regarded as an all-or-none phenomenon based on that diagnostic label. Indeed, under the terms of the MCA, retention of capacity is assumed, and capacity is both decision and time specific.

### Study design

This was a national population-based observational study examining anonymised individual-level death registration data collated by the Office for National Statistics (ONS) from 2001 to 2018, provided to us under license, and relating to heart failure decedents resident in England.

### Data source and study cohort

In the UK, the death certificate is completed by the responsible clinician, civil registration of the cause of death by a relative or another qualified informant being legally required within 5 days of medical certification. Sometimes a coroner assumes this role after a post-mortem examination or inquest. Following transcription of the information on the death certificate by the recording registrar, this is digitised and uploaded to the ONS for subsequent diagnostic coding in accordance with the 10th revision of the International Statistical Classification of Diseases and Related Health Problems (ICD-10). This study dataset comprised all deaths registered in England from January 2001 through December 2018 of people aged ≥ 65 years for whom heart failure was recorded as the underlying cause of death. We elected to study those aged ≥ 65 years at death a priori, perceiving this subset to include the vast majority of heart failure decedents with or without dementia as a marker of cognitive impairment. Heart failure as the primary cause of death was determined by the allocation of any ICD-10: I50 code by the ONS. Designation of diminished intellectual capacity for this cohort was determined when dementia of any aetiology was mentioned as a contributory cause of death. The presence of comorbid dementia was indicated by the application of ICD-10 codes G30 [Alzheimer’s disease]; F00 [dementia in Alzheimer’s disease with late onset, atypical or mixed type, and unspecified]; F01 [vascular dementia]; F02 [dementia in other diseases classified elsewhere]; or F03 [unspecified dementia]. ONS death data acquisition and coding processes are subject to regular quality assurance. Pertinent to this study period, it should be noted that in 2011 there was a change in ONS mortality data coding practice, the previous coding of unspecified cerebrovascular disease when registered as a contributory cause of death being reclassified as vascular dementia [20].

### Variables

The outcome variable was PoD as recorded on death certificates and codified by the ONS. Characterisation of PoD for this study was based on the classification system defined by the National End of Life Care Intelligence Network, part of Public Health England [21]. This specifies 5 groupings of PoD: (a) Hospital, which incorporates all acute, specialist, and community hospitals whether they be National Health Service (NHS) or private, but not psychiatric hospitals; (b) Care home, including residential and nursing homes; (c) Own residence, the decedent’s usual place of abode, but excludes communal living arrangements such as convents, monasteries, hostels or prisons; (d) Hospice, commonly standalone NHS or independent establishments; (e) Other places, covering psychiatric hospitals, other people’s homes, communal living institutions as described above, workplaces, public spaces or roads. This grouping also applies to those declared dead on arrival at hospital, potentially relevant to some heart failure patients who succumb to sudden death. Where the PoD was unknown or unspecified, these data were incorporated in descriptive statistics but not considered further in multiple adjusted analyses.

Period of death as the independent variable of interest incorporated a binary indicator for the year of death pre- and post-enforcement of the MCA [0: 2001–2007; 1: 2008–2018]. Covariates included age at death, number of mentioned contributory causes, gender, marital status, socioeconomic position as measured by the Index of Multiple Deprivation, and categorisation of the location of decedents’ usual residence as urban or rural based on the relevant postcode as archived on the ONS classification system [22, 23].

### Statistical analysis

Categorical and continuous variables were described using count (percentages) and means (standard deviation [SD]) as appropriate. The proportion of hospital deaths among heart failure decedents with or without comorbid dementia and the number of patients who died from heart failure with comorbid dementia were plotted to visually determine temporal trends, the latter also assessed statistically using a two-tailed Cochran-Armitage trend test.

We used modified Poisson regression with robust error variance [24] to evaluate the independent association between enforcement of the MCA and PoD. Three models were constructed separately for heart failure patients who died with or without comorbid dementia: home (1) versus hospital (0); care home (1) versus hospital (0); hospice (1) versus hospital (0). All covariates were forced to stay in the models to control their effects. The prevalence ratio (PR) was derived from the respective model to quantify the magnitude of association.

All analyses were performed using SAS 9.4 (SAS Institute, Cary, NC, USA). To control for Type 1 error, we applied Bonferroni correction to the alpha level. A two-sided *p* value of 0.008 (0.05/6) was considered statistically significant.

## Results

### Study sample

**Fig. 1:**
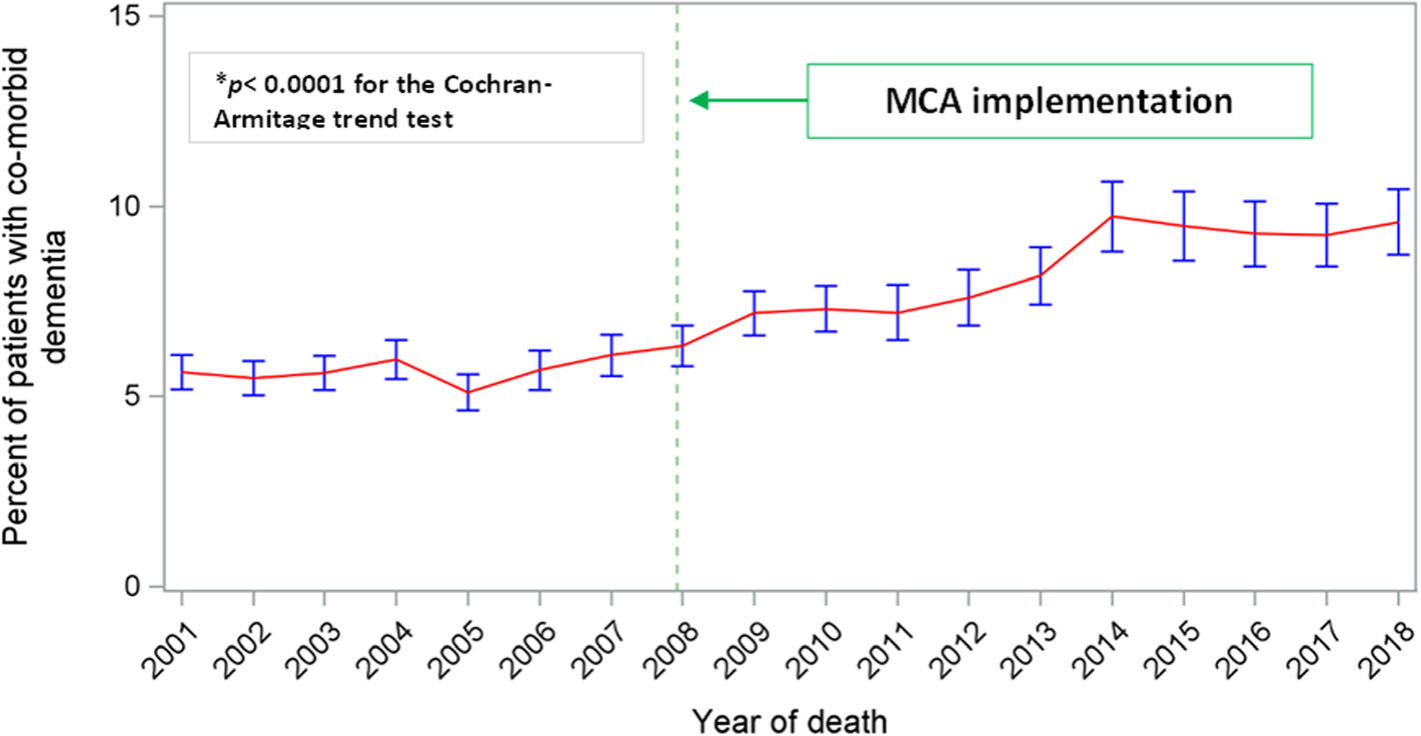
Percentages of heart failure decedents (*n*=120,068) with comorbid dementia (*n*=8199) by year of death, England, 2001–2018

Between 2001 and 2018, 120,068 people aged ≥65 years whose deaths were registered as directly due to heart failure were identified, their data subsequently included in this analysis. Table 1 describes the characteristics of these heart failure decedents. Overall, 8199 (6.8% [confidence intervals (CI) 6.7 to 7.0]) of these registrations were documented with dementia as a contributory cause, this being classified as unspecified dementia in 78.2%, Alzheimer’s disease in 13.5%, and as vascular dementia in 8.3%. No other dementia subtypes were denoted. For the periods 2001–2007 and 2008–2018, pre- and post-enforcement of the MCA, the numbers of heart failure decedents with dementia were 3427 (5.6%) and 4772 (8.0%) respectively. The prevalence proportion of dementia gradually increased on an annual basis, this trend being statistically significant (*Z*=18.87, *p*< 0.0001) as shown on Fig. 1. There was no discernible artefactual change in the general rate of dementia mentioned as a contributory cause of death associated with the 2011 change in ONS coding practice. However, as shown in Table 2, there was a contemporaneous and statistically significant reduction (*p*< 0.0001) in the coding of ‘*unspecified dementia*’ with an equivalent increase in coding for ‘*vascular dementia*’.

**Table 1:** Characteristics of heart failure decedents with or without comorbid dementia, *n* (column %), England 2001–2018

Variable	Value	With dementia	Without dementia
All	All	8199 (6.8)	111,869 (93.2)
Age at death (years)	65–74	207 (2.5)	9293 (8.3)
	75–84	2082 (25.5)	33,920 (29.9)
	85+	5910 (72.0)	68,656 (61.8)
Gender	Female	5498 (67.2)	67,474 (60.0)
	Male	2701 (32.8)	44,395 (40.0)
Marital status	Divorced	1945 (23.7)	30,118 (27.1)
	Single	365 (4.4)	5343 (4.9)
	Widowed	553 (6.8)	8481 (7.5)
	Married	5309 (64.8)	67,497 (60.1)
	Unknown	27 (0.3)	430 (0.4)
Year of death	2001–2007	3427 (87.2)	57,240 (88.5)
	2008–2018	4772 (56.4)	54,629 (55.7)
No. comorbidities	0	--	15,139 (13.4)
	1	1595 (19.4)	44,661 (39.3)
	2	3474 (42.5)	31,048 (27.9)
	3	1922 (23.4)	13,750 (12.6)
	4+	1208 (14.6)	7271 (6.8)
Deprivation*	Most deprived	1445 (17.7)	20,352 (18.1)
	2	1685 (20.5)	22,250 (19.8)
	3	1836 (22.4)	24,227 (21.7)
	4	1748 (21.3)	23,918 (21.4)
	5	1485 (18.1)	21,122 (19.0)
Rural/urban indicator*	Urban	6653 (81.1)	89,986 (80.4)
	Rural	1546 (18.9)	21,883 (19.6)
Place of death	Hospital	4033 (49.2)	73,177 (64.9)
	Care home	3497 (42.8)	21,850 (19.5)
	Home	623 (7.5)	15,541 (14.4)
	Hospice	22 (0.3)	606 (0.6)
	Other places	24 (0.3)	695 (0.6)

*p values for the difference between the two groups = 0.12. For all other inter-group comparisons, *p*< 0.0001

**Table 2:** Distribution of dementia subtypes [*n* (%)] following the ONS change in dementia coding practice of 2011

Coding	Pre- coding change	Post-coding change	Total
**AD**	642 (12.8)	465 (14.6)	1107 (13.5)
**UD**	4287 (85.5)	2128 (66.8)	6415 (78.2)
**VD**	84 (1.7)	593 (18.6)	677 (8.3)
**Total**	5013 (61.1)	3186 (38.9)	8199 (100)

*AD* Alzheimer’s disease, *UD* unspecified dementia, *VD* vascular dementia

The *p* values for comparison of the proportions of UD and VD pre- and post-coding change were statistically significant (*p*< 0.0001)

Most heart failure decedents were ≥85 years old at the time of death (62.1%). The mean age at death increased between the two study periods (2001–2007: 85.7 years [SD 7.4]; 2008–2018: 86.6 years [SD 7.5], the age at death being greater for those with dementia for both intervals at 87.1 [SD 6.2] and 88.1 years [SD 6.0], respectively. A relatively higher level of multimorbidity was noted for heart failure decedents with dementia. Most of those dying from heart failure were female, this proportionately greater at 67.2% for the dementia group compared to 60.0% for those without dementia. Across the totality of heart failure decedents, there was no significant difference in marital status between those with or without dementia, Most heart failure patients lived and died in urban environments and deprivation quintiles were similar for both study populations.

### Trends in place of death

**Fig. 2:**
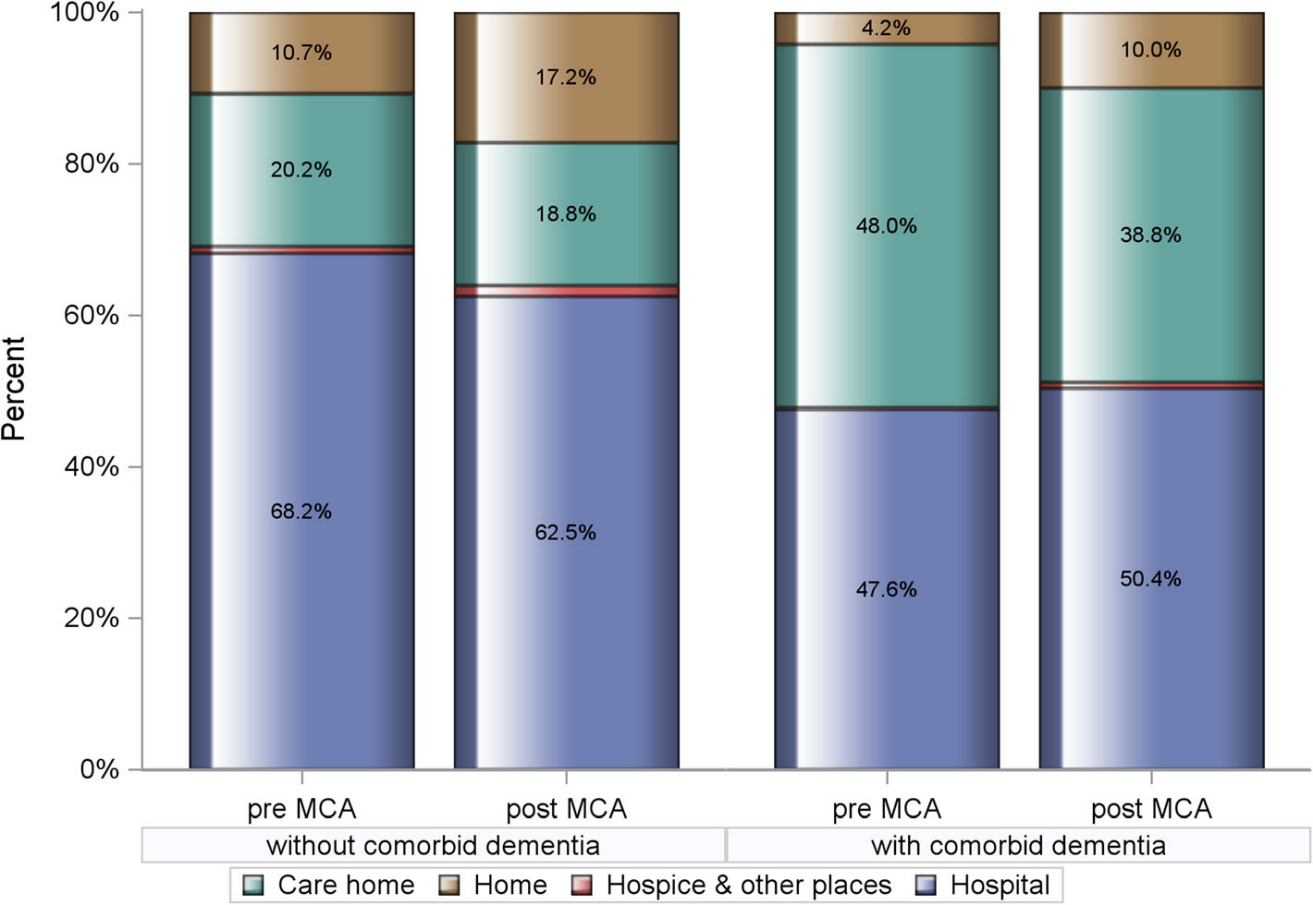
Comparative outcomes (%) in place of death for heart failure decedents with and without comorbid dementia pre- (2001–2007) and post- (2008–2018) implementation of the Mental Capacity Act (MCA). Chi-square test *p* value < 0.0001

**Fig. 3:**
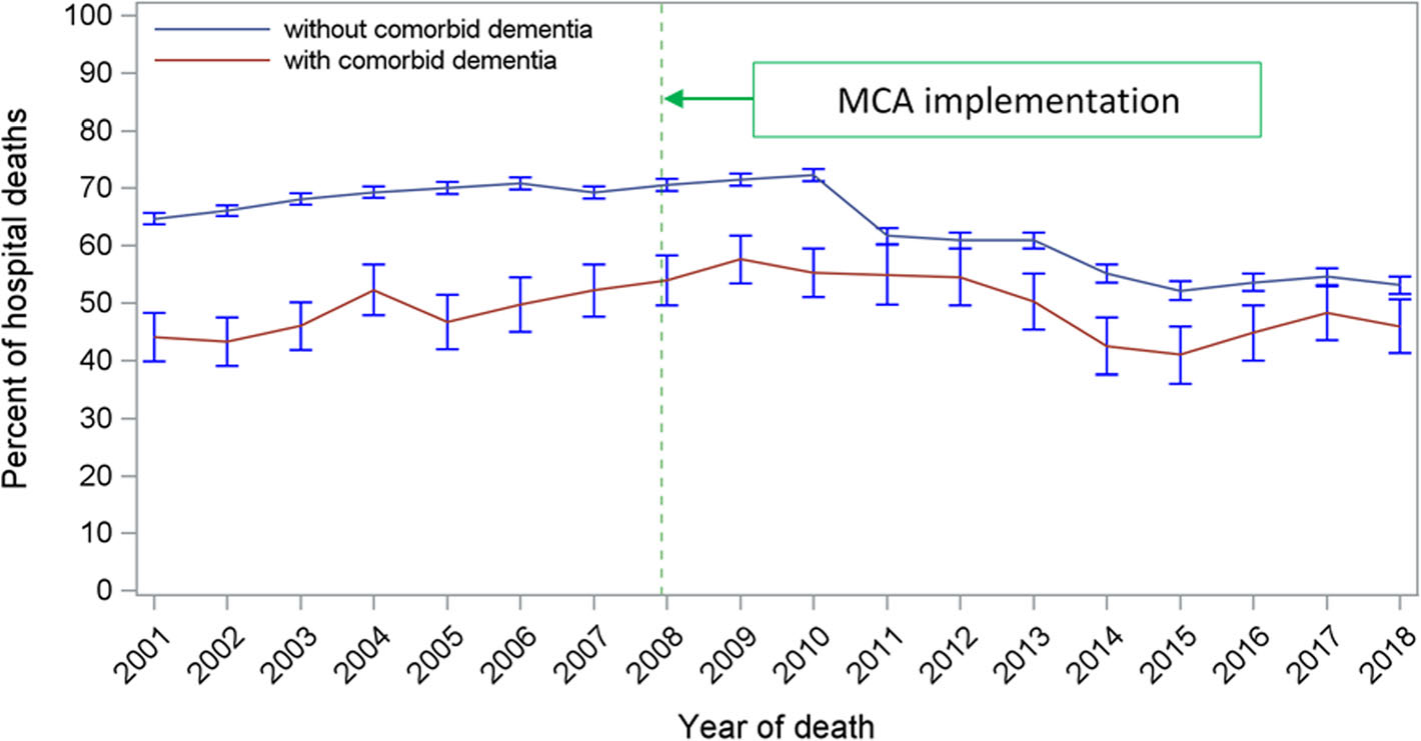
The time trend of hospital deaths among patients who died from heart failure with or without comorbid dementia, England 2001–2018

Over the period of implementation of the MCA, comparative outcomes in PoD for these heart failure decedents are shown in Fig. 2. For the period 2001–2007, hospital was the most common PoD for the non-demented heart failure group at 68.2%, 20.2% dying in a care home, and 10.7% dying at home. This changed a little for the latter period 2008–2018, reducing to 62.5% and 18.8% for hospital and care homes respectively, the proportion of home deaths increasing to 17.2%. In contrast, for heart failure decedents with dementia, there was a small increase in the proportion of hospital deaths, this rising from 47.6 to 50.4%. For this group, there was a reduction in care home deaths from 48.0 to 38.8%, with a modest rise in home deaths, 4.2% to 10.0%. Hospice as the PoD was rare for both clinical cohorts and declined over the study period. The time trend in hospital deaths is shown in Fig. 3. There was a statistically significant reduction in hospital deaths for heart failure decedents without dementia (*p*< 0.001). On the other hand, the marginal increase in hospital deaths for those with dementia was not significant (*p*=0.97). Adjusted PRs following implementation of the MCA confirm increased PoD at home compared to hospital for non-dementia patients, PR: 1.026 [CI: 1.024–1.029] (*p*< 0.0001), this trend not significant for those with dementia, PR: 1.001 [CI 0.988–1.015] (*p*=0.83). Care home deaths reduced for both groups, PR: 0.959 [CI 0.949–0.969] (*p*< 0.0001), and PR: 0.995 [CI 0.993–0.998] (*p*< 0.0001) for those with and without dementia, respectively. Starting from an already small base, adjusted PRs for hospice rather than hospital as PoD declined significantly for both non-demented and demented heart failure decedents being 0.979 [CI 0.977–0.980 (*p*< 0.0001) and 0.946 [CI 0.934–0.959] (*p*< 0.0001), respectively. A summary of the adjusted PRs for dying in a premise other than hospital following MCA enforcement is shown in Table 3. Fully detailed results for all three model sets are available in Additional file 1: Supplementary Tables S-2 to S-4.

**Table 3:** The adjusted prevalence ratios* (95% confidence intervals) of dying in a specific type of premise (compared to a hospital death) after implementation of MCA in heart failure decedents with or without comorbid dementia, England 2001–2018

	With dementia		Without dementia	
Home	1.001 (0.988 to 1.015)	*p*=0.83	1.026 (1.024 to 1.029)	*p*< 0.0001
Care home	0.959 (0.949 to 0.969)	*p*< 0.0001	0.995 (0.993 to 0.998)	*p*< 0.0001
Hospice	0.946 (0.934 to 0.959)	*p*< 0.0001	0.979 (0.977 to 0.980)	*p*< 0.0001
Hospital (Reference)	1.000		1.000	

*> 1 indicates a higher chance of death in the corresponding type of premise

## Discussion

In this first national study examining variation in the PoD for heart failure patients over the period of enactment and implementation of the MCA in England, our results suggest that the decision-making and advance care planning processes enshrined in this legislation had little material effect on the ultimate site of care provision determining PoD for those with cognitive impairment manifest as dementia. In the later years of this study period when the code of practice for this legislation was operational, trend data for heart failure decedents certified with comorbid dementia show a modest rise in hospital deaths with fewer care home deaths. Conversely, for this study phase, there was a small reduction in hospital deaths for those without dementia who were younger and with fewer comorbidities.

The reasons behind the apparent lack of impact of this legislation may be complex, but it has been proposed that the MCA is relatively poorly applied in clinical practice. While a variety of training models have been developed to disseminate information to health professionals on the five principles underpinning these regulations, recent reviews suggest poor understanding of when and how the provisions of the Act should be employed, with the need for clarity on the process of designating the role of surrogate decision-makers to properly ensure patients’ best interests are maintained [25–27]. A lack of confidence of those working in acute care settings has been particularly highlighted, specifically citing decision-making with respect to hospital discharge [28]. This issue may be especially relevant to our study observations given that we have demonstrated that the majority of those dying with heart failure in England do so in hospital, similar data emerging from the United States (US) [29].

At variance with the trends evident in this study, previous work has suggested a decline in the frequency of hospital deaths for those with dementia in recent years, with more people dying in care homes [30]. These findings were also based on ONS death certification data but included all individuals for whom dementia was mentioned as either the underlying or as a contributory cause of death. In contrast, the current study specifies heart failure as the primary cause of death, differentiating the comparator groups by the presence or absence of dementia mentioned only as a contributory cause. It is well established that the principal diagnosis is the main determinant of the site of clinical care [31], and community-based primary care practitioners appear to be relatively incognizant of patients’ preferences for place of care or PoD, particularly when dealing with non-cancer diagnoses [32]. Unless policies for comfort care are clearly outlined, should people with chronic heart failure living at home or in a nursing home suddenly deteriorate, the reactive response of professional staff may be to arrange emergency hospital admission by default. However, we have no information on any care transitions prior to the terminal phase for this study cohort, or whether this possible course of action had a bearing on the results of this study.

The completeness of death certification in the UK is regarded as relatively robust with proportionately fewer ‘*garbage codes*’ than data from many other countries [33]. However, dementia as recorded on death certification likely underestimates the true prevalence, and it has been suggested that studies using death certification alone may fail to account for 16-18% of dementia cases [34]. A variety of factors may influence such documentation. Rates of inclusion of dementia are generally increased in those who die in institutions such as care homes compared to those dying at home, particularly if dementia is at the severe end of the clinical spectrum and includes agitation [35]. While heart failure guidelines draw attention to cognitive impairment as a comorbidity, describing all grades of this by hospital-based clinicians is reportedly poor [14], and there are diverging views on whether dying in hospital positively or negatively affects the rate of recording of dementia at the time of death certification [35, 36]. Recent initiatives to heighten clinicians’ awareness of dementia may improve matters. In 2012, NHS England introduced a quality improvement scheme through the Commissioning for Quality and Innovation (CQUIN) payment framework [37]. Acute healthcare providers were incentivised with the assurance of increased remuneration if 90% of all patients aged ≥75 years and whose emergency hospital admission lasted > 72 h were screened for dementia. Further financial gain was available if those patients whose initial assessment indicated dementia or was inconclusive were referred on for specialist review. While this dementia assessment and referral exercise was retired as a CQUIN indicator in April 2016, these conditions have been retained within the standard contract for English hospitals providing acute clinical services. It is possible that these administrative processes may have contributed in some measure to the increased mentions of dementia as certified for hospital decedents evident in the latter course of this study.

The relatively frequent concurrence of cognitive impairment and heart failure likely stems from various pathophysiologic features related to the latter condition combined with shared cardiovascular risk factors such as hypertension, hyperlipidaemia or dysglycaemia [7, 38, 39]. The haemodynamic and risk factor profiles for HFrEF and HFpEF clearly differ, but very few investigations have compared the spectrum of cognitive impairment across the range of ejection fraction phenotypes. There is a suggestion that affected domains of cognitive function may vary, but data is limited with inconsistent results [40, 41].

To date, there is no evidence that evidence-based guideline-directed medical therapy (GDMT) for heart failure drives neurocognitive dysfunction [42], and indeed it has been posited that centrally acting angiotensin-converting enzyme inhibitors (ACEIs) such as perindopril or captopril, which cross the blood-brain barrier, may slow the progression of cognitive impairment in those with dementia [43]. Following the positive results of the PARADIGM-HF study demonstrating the benefits of sacubitril/valsartan, the first of a new class of drugs termed ARNIs (angiotensin receptor-neprilysin inhibitors) [44], this therapeutic option for HFrEF has been widely adopted. Neprilysin is a soluble metalloprotease which catalyses the degradation of natriuretic peptides (NPs), downregulation of this enzymatic activity likely increasing endogenous NP mediated natriuresis and vasodilation. However, such neprilysin inhibition might also interfere with the clearance of amyloid-β protein, vascular deposition of which results in cerebral amyloid angiopathy, a distinctive feature of Alzheimer’s disease. Dementia-related adverse events were not overrepresented through 4.3 years follow-up of the relevant PARADIGM-HF study arm compared to similar populations [45]. Nonetheless, as required by the Food and Drug Administration in the US, this potential hazard is currently being evaluated in the PERSPECTIVE study (ClinicalTrials.gov ID NCT02884206). Due to report in 2022, this trial includes a battery of neurocognitive testing and sequential ^18^F-labelled florbetaben positron emission tomography to assess any longitudinal changes in cerebral amyloid plaque burden. Importantly, recent evidence shows that the hearts of some patients with Alzheimer’s disease exhibit diastolic dysfunction and thickening of the interventricular septum. These features are characteristic of cardiac amyloidosis suggesting that in some individuals, amyloid-β protein may also accumulate in tissues other than the brain [46].

As inferred above, in recent years GDMT for those affected by heart failure has become increasingly effective [47], but heart failure is an ambulatory care sensitive condition and remains the commonest cause of acute hospitalisation in those > 65 years [48]. Following an index heart failure admission in England, the 1-year mortality for patients discharged alive is 39.6% with a 30-day all-cause readmission rate of 19.8% [49]. Readmissions for heart failure tend to follow a tri-phasic pattern. This was apparent in a study of 8543 heart failure patients in Toronto monitored for 10 years following their first hospital admission, by which time 98.8% had died, the median survival after heart failure diagnosis being 1.75 years [50]. About 30% of all readmissions occurred within 2-months of initial hospital discharge, 50% during the 2-month period leading up to death, with 15-20% taking place in the intervening ‘*plateau phase*’ of the heart failure disease trajectories. A sentinel clustering of admissions in the terminal phase of heart failure has been well described [51]. It is uncertain if the presence of dementia as a comorbidity influences the readmission rate. Rao and colleagues followed 10,317 patients for 5 years subsequent to their diagnosis with heart failure between April 2008 and March 2009 using the primary care-based Clinical Practice Research Datalink combined with Hospital Episode Statistics and ONS death registration data [52]. Their analysis indicated that comorbid dementia was a factor significantly affecting emergency hospital readmissions in only 3 of 8 regions across England.

Comparable to the epidemiological trends for heart failure, the age-adjusted prevalence and incidence of dementia may also be declining in high-income countries. The Medical Research Council Cognitive Function and Ageing Studies (CFAS 1 and II) of populations living in rural Cambridgeshire and the urban environments of Newcastle and Nottingham demonstrated a 24% reduction in the prevalence of dementia in those ≥65 years between 1989 and 2011 [53]. Consistent with our observations, the CFAS studies also suggested that women were more commonly affected, and while the prevalence of dementia in care home residents had increased from 56 to 70%, most people with dementia were still living at home. Similarly, dementia events have been continuously surveyed in the US-based Framingham Heart Study since 1975. Monitoring of this community cohort living in Massachusetts, predominantly of white European ancestry, has implied a 20% stepwise decline in the incidence of dementia each decade over the last 30 years [54]. The background to these cumulative decrements remains to be determined, but both the CFAS and Framingham study groups cited potential mechanisms in higher early educational attainment and attenuated vascular morbidity.

The Framingham Heart Study showed a non-significant reduction in Alzheimer’s disease with a more overt decrease in vascular dementia. In Westernised societies, Alzheimer’s is the most commonly encountered manifestation of dementia, but as an isolated pathophysiological process, this affects < 20% of those with heart failure. Rather, vascular dementia has been proposed as the likeliest associated variant, followed by mixed forms, then Alzheimer’s and other specific dementias [39]. This is at odds with the distribution of dementia subtypes noted in this study where unspecified dementia was most frequently mentioned on death certificates and coded as the dominant category. A similar finding was described in a Danish study of 324,418 patients admitted with incident heart failure and tracked for 35 years against an age- and sex-matched population without heart failure selected from the Danish Civil Registration System [55]. Adelborg and colleagues found a clear association between all-cause dementia and heart failure. This was relatively weak for Alzheimer’s disease, and while the vascular variant was represented, this was predominantly determined by the reported development of unspecified dementia. These authors proposed that some patients ostensibly exhibiting unspecified dementia may have been misclassified. However, in combining death certification data from sequential CFAS studies in England, all mentions of dementia as the underlying or a contributory cause of death showed a percentage distribution of subtypes of unspecified dementia, Alzheimer’s disease and vascular dementia as 69.3%, 21.6% and 8.6%, respectively [37]. These results are very similar to those noted in the current study and suggest that this proportional distribution of dementia variants is not specific to the heart failure population.

Advance care planning offers patients the potential to receive medical treatment consistent with their expressed preferences, values, and goals of care against the possibility of subsequent loss of decision-making capacity. This may help prevent needless hospital admissions and better achieve consensus on appropriate ceilings of care, avoiding exposure of patients and families to the distressing harms which sometimes accompany futile treatment escalation and burdensome invasive interventions close to the end of life. It is notable that a recent audit of end-of-life care in hospitals in England demonstrated that10% of heart failure patients were receiving mechanical ventilatory support within 24 h of death [56].

Given the unpredictability intrinsic to the heart failure disease trajectory which challenges individual prognostication even in the late stages of this disease, and the associated multimorbidity including cognitive impairment, it might be expected that advance care planning would be central to the care of people with this condition. However, advance care planning is not routinely incorporated within heart failure care and tends to be limited to the possible withdrawal of any implanted electronic or mechanical devices, or sometimes offered as one component of the still uncommon provision of palliative care [57, 58]. A review and meta-analysis of 14 randomised controlled trials of advance care planning in heart failure, mostly US-based and involving 2924 individuals across a range of care structures, showed this to moderately improve the primary outcome measure in patients’ quality of life, together with similarly weighted favourable effects on secondary outcomes including communication about, and satisfaction with, end-of-life care [59].

Advance care planning for dementia was featured as a specific domain in the European Association of Palliative Care white paper on this condition [60], and the challenges taking this forward have been comprehensively reviewed [61, 62]. Emerging themes suggest that while there is some disparity in the readiness of older people to engage in advance care planning, and the means to take this forward may also vary across the spectrum of healthcare delivery, the use of these instruments may be effective in promoting shared decision-making between patients, informal carers and professionals [63]. However, it should be noted that, in the UK, an advance care plan is not legally binding but merely an advisory statement of preferences and wishes in relation to general care and medical therapy [64].

Dialogue between the patient, family and clinician is basic to shared decision-making. However, triangulation of information between this triad does not necessarily mean equitably weighted opinions, and at times patients’ voices are marginalised, a dyadic interchange conducted between clinicians and family members. Such a discourse may be justified if the patient cannot physically contribute to meaningful shared decision-making, if capacity is deemed to be lost, legally binding instruments are not in place, and their preferences are unknown. Then, the opinion of the closest relative should be sought, their views respected and used to inform a care plan constructed in the best interests of the patient. However, it should also be remembered that the involvement of relatives as decisional proxies may be emotionally demanding, particularly if they are already distressed and experiencing anticipatory grief. Further, the assumption that there is always congruence between the opinions of family surrogate decision-makers and those perceived of patients is flawed. Rather, these are often misaligned, with at best moderate concordance between dementia/carer dyads when assessed within a hospital setting [65]. Qualitative studies examining difficulties in decision making during clinical encounters for those with dementia have cited tension between family members and conflicts between families and health professionals, some of the latter reluctant to undertake decisions on patients unfamiliar to them, particularly when there is poor information exchange following transfer from another care setting [66]. Even if decisional consensus is achieved, the discharge of hospital inpatients home may be subject to practical limitations depending on the social context. The caregiver burdens associated with heart failure and dementia have been well characterised. Informal caregivers may be unwilling or unable to reframe or enhance their roles, and it should be noted that the cohabitees of people living and dying with dementia tend to be of a similar advanced age [67]. Further, it has been proposed that there may be a gendering issue relating to care at home in that older women are more likely to have outlived their male partners and be devoid of spousal support [68]. However, in this study, the marital status of heart failure decedents with or without dementia appeared to be similar. Following the implementation of the MCA, our analysis suggests that proportionately more women with dementia died at home compared to those without dementia. The reason for this disparity is unclear, and a variety of other intersectional stressors which might constrain the social capital of older women close to the end of life may have been in play [69, 70].

### Strengths and weaknesses

To our knowledge, this is the first empirical study to systematically examine national data for England describing the PoD of people dying of heart failure with dementia documented as a contributory cause of death. A major strength is that our work is based on comprehensive data collated from the gamut of clinical practice over an 18-year period, but we acknowledge that we are dependent on the clinicians responsible for these heart failure decedents having made the correct diagnoses and accurately completing later death certification, over which there is no means of independent adjudication. Irrespective of the PoD, we have no access to information on the underlying aetiologies of heart failure, or the proportional distribution of resultant ejection fraction variants. Similarly, it is not possible to ascertain the duration of heart failure or nature of any care provision prior to the terminal phase, and whether this fatal outcome related to incident acute heart failure, worsening of chronic heart failure, or heart failure-related sudden cardiac death. It has been suggested that decisions to include dementia on death certificates rely on medical staff regarding this as clinically significant [71]. This is considered more likely if dementia is relatively severe, reaffirming our judgement in using the certified mention of dementia as a contributory cause of death to represent a valid marker of significant cognitive dysfunction, and therefore relevant to the aegis of the MCA. Regarding generalisability, the results of this study are germane to similar clinical populations, models of care delivery, and legal constructs. While the legal status of the MCA 2005 applies to England and Wales, beyond this jurisdiction but within the UK, this legislation is closely aligned to the Adults with Incapacity (Scotland) Act 2000, and the Mental Capacity Act (Northern Ireland) 2016. There is also some global resonance through Article 12 of the United Nations Convention on the Rights of Persons with Disabilities (CRPD) of 2006. While the pertinence of specific aspects of the CRPD have been subject to legal argument [72], both this and the MCA have informed the development of mental capacity policies and legislation in Canada, Australia, and New Zealand [73, 74].

## Conclusions

In this hypothesis-generating study, we have investigated the impact of the MCA on the PoD of heart failure decedents aged ≥65 years resident in England whom we have shown to be increasingly affected by comorbid dementia, dying at home or usual place of residence customarily accepted as the preferred option. Our analyses of trends over the period of enactment and implementation of the code of practice relating to this legislation show little to suggest any significant influence on PoD for this relatively vulnerable clinical cohort. The background to this somewhat neutral outcome is multifaceted, administration of the Act clearly challenging for a prognostically ambiguous population subject to flux in the often-nuanced scenarios typical of real-life clinical practice, a milieu dominated by the treatment imperative. Further, even if the precepts of the MCA are correctly applied, this course of action may be ineffectual in isolation. Recent evidence suggests that achieving a good death at home requires patients and their informal carers to feel secure in that setting, effected by the provision of a 24/7 responsive palliative care service, staffed by those competent in symptom relief and with good communication skills [75]. Fulfilling the preferred PoD of those with heart failure and dementia might be better achieved by embedding application of the MCA within a system of anticipatory sympathetic clinical navigation across all care sectors, contingent upon effective upskilling of the relevant professionals, with good inter-agency and multidisciplinary collaboration to support and maintain appropriately configured community-based palliative care.

## Supplementary Information

**Additional file 1.** Supplementary Table S-1. Characteristics of heart failure decedents, with or without comorbid dementia, before and after MCA implementation, n (column %), England 2001-2018. Supplementary Table S-2. Multiple adjusted prevalence ratios for the factors associated with home death compared to hospital death among patients who died from heart failure with or without dementia, England 2001-2018. Supplementary Table S-3. Multiple adjusted prevalence ratios for the factors associated with care home death compared to hospital death among patients who died from heart failure with or without dementia, England 2001-2018. Supplementary Table S-4. Multiple adjusted prevalence ratios for the factors associated with hospice death compared to hospital death among patients who died from heart failure with or without dementia, England 2001-2018

## Data Availability

Additional file 1:Supplementary files S-1, S-2, S-3, and S-4 are available online.

## Abbreviations

ACEI: Angiotensin-converting enzyme inhibitor
ARNI: Angiotensin receptor-neprilysin inhibitor
CFAS: Cognitive Function and Ageing Study
CQUIN: Commissioning for Quality and Innovation
EF: Ejection fraction
GDMT: Guideline directed medical therapy
HF: Heart failure
HFimpEF: Heart Failure with improved Ejection Fraction
HFmrEF: Heart Failure with mildly reduced Ejection Fraction
HFpEF: Heart Failure with preserved Ejection Fraction
HFrEF: Heart Failure with reduced Ejection Fraction
ICD-10: International Classification of Diseases 10^th^ Revision
ID: Identifier
LPA: Lasting Power of Attorney
MCA: Mental Capacity Act
NHS: National Health Service
NP: Natriuretic peptide
ONS: Office for National Statistics
PoD: Place of death
SD: Standard deviation
UK: United Kingdom
US: United States
