# Supplementary material for: Implementation of the Mental Capacity Act: a national observational study comparing resultant trends in place of death for older heart failure decedents with or without comorbid dementia
Source: BMC Med. 2022 Jan 20;20:30. doi: 10.1186/s12916-021-02210-2 (PMC9901524; doi:10.1186/s12916-021-02210-2)
Supplement: Supplementary file 1 — Additional file 1. Supplementary Table S-1. Characteristics of heart failure decedents, with or without comorbid dementia, before and after MCA implementation, n (column %), England 2001-2018. Supplementary Table S-2. Multiple adjusted prevalence ratios for the factors associated with home death compared to hospital death among patients who died from heart failure with or without dementia, England 2001-2018. Supplementary Table S-3. Multiple adjusted prevalence ratios for the factors associated with care home death compared to hospital death among patients who died from heart failure with or without dementia, England 2001-2018. Supplementary Table S-4. Multiple adjusted prevalence ratios for the factors associated with hospice death compared to hospital death among patients who died from heart failure with or without dementia, England 2001-2018 [file 12916_2021_2210_MOESM1_ESM.docx]

**Supplementary Table S-1.** Characteristics of heart failure decedents, with or without comorbid dementia,

before and after MCA implementation, n (column %), England 2001-2018.

| Variable  MCA implementation | Value | With dementia | | | Without dementia | | |
| --- | --- | --- | --- | --- | --- | --- | --- |
|  |  | Before | After | All | Before | After | All |
| All | -- | 3929 (47.9) | 4270 (52.1) | 8199 | 64664 (57.8) | 47205 (42.2) | 11869 |
| Age | 65-74 | 107 (2.7) | 100 (2.3) | 207 (2.5) | 5487 (8.5) | 3806 (8.1) | 9293 (8.3) |
|  | 75-84 | 1114 (28.4) | 968 (22.7) | 2082 (25.5) | 20923 (32.4) | 12997 (27.5) | 33920 (29.9) |
|  | 85+ | 2708 (68.9) | 3202 (75.0) | 5910 (72.0) | 38254 (59.2) | 30402 (64.4) | 68656 (61.8) |
| Gender | Female | 2752 (70.0) | 2746 (64.3) | 5498 (67.2) | 40238 (62.2) | 27236 (57.7) | 67474 (60.0) |
|  | Male | 1177 (30.0) | 1524 (35.7) | 2701 (32.8) | 24426 (37.8) | 19969 (42.3) | 44395 (40.0) |
| Marital status | Married | 863 (22.0) | 1082 (25.3) | 1945 (23.7) | 16629 (25.7) | 13489 (28.6) | 30118 (27.1) |
|  | Divorced | 147 (3.7) | 218 (5.1) | 365 (4.4) | 2604 (4.0) | 2739 (5.8) | 5343 (4.9) |
|  | Single | 301 (7.7) | 252 (5.9) | 553 (6.8) | 5355 (8.3) | 3126 (6.6) | 8481 (7.5) |
|  | Widowed | 2607 (66.4) | 2702 (63.3) | 5309 (64.8) | 39825 (61.6) | 27672 (58.6) | 67497 (60.1) |
| No. comorbidity | 0 | -- | -- | -- | 9370 (14.5) | 5769 (12.2) | 15139 (13.4) |
|  | 1 | 751 (19.1) | 844 (19.8) | 1595 (19.4) | 27982 (43.3) | 16679 (35.3) | 44661 (39.3) |
|  | 2 | 1830 (46.6) | 1644 (38.5) | 3474 (42.5) | 17311 (26.8) | 13737 (29.1) | 31048 (27.9) |
|  | 3 | 896 (22.8) | 1026 (24.0) | 1922 (23.4) | 6812 (10.5) | 6938 (14.7) | 13750 (12.6) |
|  | 4+ | 452 (11.5) | 756 (17.7) | 1208 (14.6) | 3189 (4.9) | 4082 (8.6) | 7271 (6.8) |
| Deprivation | Most deprived | 725 (18.5) | 720 (16.9) | 1445 (17.7) | 12128 (18.8) | 8224 (17.4) | 20352 (18.1) |
|  | 2 | 804 (20.5) | 881 (20.6) | 1685 (20.5) | 13087 (20.2) | 9163 (19.4) | 22250 (19.8) |
|  | 3 | 905 (23.0) | 931 (21.8) | 1836 (22.4) | 14019 (21.7) | 10208 (21.6) | 24227 (21.7) |
|  | 4 | 828 (21.1) | 920 (21.5) | 1748 (21.3) | 13621 (21.1) | 10297 (21.8) | 23918 (21.4) |
|  | 5 | 667 (17.0) | 818 (19.2) | 1485 (18.1) | 11809 (18.3) | 9313 (19.7) | 21122 (19.0) |
| Settlement | Urban | 3207 (81.6) | 3446 (80.7) | 6653 (81.1) | 51862 (80.2) | 38124 (80.8) | 89986 (80.4) |
|  | Rural | 722 (18.4) | 824 (19.3) | 1546 (18.9) | 12802 (19.8) | 9081 (19.2) | 21883 (19.6) |
| Place of death | Hospital | 1901 (48.4) | 2132 (49.9) | 4033 (98.3) | 44260 (68.4) | 28917 (61.3) | 73177 (129.7) |
|  | Care home | 1844 (46.9) | 1653 (38.7) | 3497 (85.6) | 12771 (19.7) | 9079 (19.2) | 21850 (39.0) |
|  | Home | 174 (4.4) | 449 (10.5) | 623 (14.9) | 7047 (10.9) | 8494 (18.0) | 15541 (28.9) |
|  | Hospice | --* | --* | 22 (0.5) | 204 (0.3) | 402 (0.9) | 606 (1.2) |
|  | Elsewhere | --* | --* | 24 (0.6) | 382 (0.6) | 313 (0.7) | 695 (1.3) |

--*: suppressed as small number below disclosure threshold of ONS as data provider.

**Supplementary Table S-2.** Multiple adjusted prevalence ratios for the factors associated with home death compared to hospital death among patients who died from heart failure with or without dementia, England 2001-2018

| **Variable** | **Value** | **With dementia** | | **Without dementia** | |
| --- | --- | --- | --- | --- | --- |
|  |  | PR (95%CI) | p value | PR (95%CI) | p value |
| Year of death  Ref: before MCA | After MCA | 1.001 (0.988 to 1.015) | 0.83 | 1.026 (1.024 to 1.029) | <0.0001 |
| Age at death  Ref: 65-74 | 75-84 | 1.025 (0.980 to 1.072) | 0.35 | 0.989 (0.986 to 0.993) | <0.0001 |
|  | 85+ | 1.015 (0.971 to 1.061) |  | 0.990 (0.987 to 0.994) |  |
| Sex  Ref: male | Female | 1.022 (1.006 to 1.038) | 0.007 | 0.997 (0.994 to 0.999) | 0.004 |
| Marital status  Ref: married | Divorced | 0.967 (0.934 to 1.001) | <0.0001 | 0.987 (0.981 to 0.992) | <0.0001 |
|  | Single | 0.930 (0.901 to 0.961) |  | 0.973 (0.969 to 0.978) |  |
|  | Widowed | 0.960 (0.943 to 0.977) |  | 0.986 (0.984 to 0.989) |  |
|  | NS/unknown | 0.964 (0.878 to 1.059) |  | 0.991 (0.973 to 1.009) |  |
| No. comorbidities  Ref: no comorbidity | 1 | 1.027 (1.000 to 1.054) | 0.18 | 0.963 (0.959 to 0.967) | <0.0001 |
|  | 2 | 1.012 (0.996 to 1.028) |  | 0.941 (0.937 to 0.945) |  |
|  | 3 | 1.004 (0.987 to 1.021) |  | 0.924 (0.920 to 0.928) |  |
|  | 4+ | 1.000 (1.000 to 1.000) |  | 0.906 (0.902 to 0.910) |  |
| Index of multiple deprivation  Ref: least deprived | Most deprived | 0.994 (0.970 to 1.018) | 0.46 | 0.994 (0.990 to 0.997) | 0.001 |
|  | 2 | 0.993 (0.969 to 1.017) |  | 0.992 (0.989 to 0.996) |  |
|  | 3 | 1.006 (0.984 to 1.030) |  | 0.995 (0.991 to 0.998) |  |
|  | 4 | 1.010 (0.987 to 1.034) |  | 0.996 (0.992 to 0.999) |  |
| Rural/urban indicator  Ref: urban | Rural | 1.014 (0.994 to 1.034) | 0.17 | 1.020 (1.017 to 1.024) | <0.0001 |

**Supplementary Table S-3.** Multiple adjusted prevalence ratios for the factors associated with care home death compared to hospital death among patients who died from heart failure with or without dementia, England 2001-2018

| **Variable** | **Value** | **With dementia** | | **Without dementia** | |
| --- | --- | --- | --- | --- | --- |
|  |  | PR (95%CI) | p value | PR (95%CI) | p value |
| Year of death  Ref: before MCA | After MCA | 0.959 (0.949 to 0.969) | <0.0001 | 0.995 (0.993 to 0.998) | <0.0001 |
| Age at death  Ref: 65-74 | 75-84 | 1.023 (0.985 to 1.064) | <0.0001 | 1.016 (1.012 to 1.019) | <0.0001 |
|  | 85+ | 1.054 (1.014 to 1.095) |  | 1.066 (1.063 to 1.070) |  |
| Sex  Ref: male | Female | 1.024 (1.011 to 1.037) | <0.0001 | 1.021 (1.019 to 1.024) | <0.0001 |
| Marital status  Ref: married | Divorced | 1.023 (0.995 to 1.053) | 0.002 | 1.026 (1.021 to 1.032) | <0.0001 |
|  | Single | 1.030 (1.005 to 1.056) |  | 1.048 (1.043 to 1.053) |  |
|  | Widowed | 1.030 (1.016 to 1.046) |  | 1.034 (1.031 to 1.037) |  |
|  | NS/unknown | 1.017 (0.932 to 1.109) |  | 1.021 (1.002 to 1.039) |  |
| No. comorbidities  Ref: no comorbidity | 1 | 1.111 (1.091 to 1.132) | <0.0001 | 0.998 (0.994 to 1.002) | <0.0001 |
|  | 2 | 1.073 (1.057 to 1.089) |  | 0.974 (0.970 to 0.978) |  |
|  | 3 | 1.047 (1.030 to 1.064) |  | 0.950 (0.945 to 0.954) |  |
|  | 4+ | 1.000 (1.000 to 1.000) |  | 0.932 (0.927 to 0.936) |  |
| Index of multiple deprivation  Ref: least deprived | Most deprived | 0.982 (0.963 to 1.000) | <0.0001 | 0.976 (0.972 to 0.980) | <0.0001 |
|  | 2 | 0.993 (0.975 to 1.011) |  | 0.988 (0.984 to 0.992) |  |
|  | 3 | 1.013 (0.996 to 1.030) |  | 0.997 (0.993 to 1.000) |  |
|  | 4 | 1.016 (0.999 to 1.034) |  | 1.000 (0.996 to 1.003) |  |
| Rural/urban indicator  Ref: urban | Rural | 1.008 (0.994 to 1.022) | 0.27 | 1.018 (1.015 to 1.021) | <0.0001 |

**Supplementary Table S-4.** Multiple adjusted prevalence ratios for the factors associated with hospice death compared to hospital death among patients who died from heart failure with or without dementia, England 2001-2018

| **Variable** | **Value** | **With dementia** | | **Without dementia** | |
| --- | --- | --- | --- | --- | --- |
|  |  | PR (95%CI) | p value | PR (95%CI) | p value |
| Year of death  Ref: before MCA | After MCA | 0.946 (0.934 to 0.959) | <0.0001 | 0.979 (0.977 to 0.980) | <0.0001 |
| Age at death  Ref: 65-74 | 75-84 | 1.016 (0.975 to 1.059) | 0.21 | 1.001 (0.999 to 1.003) | <0.0001 |
|  | 85+ | 1.004 (0.964 to 1.046) |  | 0.995 (0.993 to 0.997) |  |
| Sex  Ref: male | Female | 1.008 (0.994 to 1.023) | 0.25 | 1.000 (0.998 to 1.001) | 0.73 |
| Marital status  Ref: married | Divorced | 1.002 (0.969 to 1.035) | 0.74 | 0.997 (0.993 to 1.000) | 0.22 |
|  | Single | 0.984 (0.955 to 1.014) |  | 1.000 (0.997 to 1.003) |  |
|  | Widowed | 0.992 (0.976 to 1.008) |  | 0.999 (0.997 to 1.000) |  |
|  | NS/unknown | 1.014 (0.933 to 1.103) |  | 0.993 (0.980 to 1.006) |  |
| No. comorbidities  Ref: no comorbidity | 1 | 0.901 (0.876 to 0.926) | <0.0001 | 1.011 (1.008 to 1.014) | <0.0001 |
|  | 2 | 0.955 (0.941 to 0.969) |  | 1.016 (1.012 to 1.019) |  |
|  | 3 | 0.971 (0.956 to 0.986) |  | 1.021 (1.018 to 1.025) |  |
|  | 4+ | 1.000 (1.000 to 1.000) |  | 1.025 (1.021 to 1.028) |  |
| Index of multiple deprivation  Ref: least deprived | Most deprived | 1.011 (0.989 to 1.034) | 0.35 | 1.002 (1.000 to 1.005) | 0.07 |
|  | 2 | 0.998 (0.976 to 1.021) |  | 1.003 (1.000 to 1.005) |  |
|  | 3 | 1.016 (0.994 to 1.038) |  | 1.002 (0.999 to 1.004) |  |
|  | 4 | 1.013 (0.991 to 1.036) |  | 1.000 (0.997 to 1.002) |  |
| Rural/urban indicator  Ref: urban | Rural | 0.990 (0.972 to 1.009) | 0.31 | 1.003 (1.001 to 1.005) | 0.003 |
